# Preclinical evaluation of binimetinib (MEK162) delivered via polymeric nanocarriers in combination with radiation and temozolomide in glioma

**DOI:** 10.1007/s11060-019-03365-y

**Published:** 2019-12-24

**Authors:** Fatima Bikhezar, Robin M. de Kruijff, Astrid J. G. M. van der Meer, Guzman Torrelo Villa, Susanne M. A. van der Pol, Gabriel Becerril Aragon, Ana Gasol Garcia, Ravi S. Narayan, Helga E. de Vries, Ben J. Slotman, Antonia G. Denkova, Peter Sminia

**Affiliations:** 1 Department of Radiation Oncology, Amsterdam University Medical Centers, Location VUmc & Cancer Center Amsterdam, De Boelelaan 1117, 1081 HV, Amsterdam, The Netherlands; 2 Department of Molecular Cell Biology and Immunology, Amsterdam University Medical Centers, Location VUmc, Amsterdam, The Netherlands; 3 Department of Radiation Science and Technology, Delft University of Technology, Delft, The Netherlands

**Keywords:** Glioblastoma, Polymeric nanocarriers, Binimetinib, Radiation, Temozolomide, Blood–brain barrier

## Abstract

**Background and purpose:**

Glioblastoma multiforme (GBM) is the most aggressive subtype of malignant gliomas, with an average survival rate of 15 months after diagnosis. More than 90% of all GBMs have activating mutations in the MAPK/ERK pathway. Recently, we showed the allosteric MEK1/2 inhibitor binimetinib (MEK162) to inhibit cell proliferation and to enhance the effect of radiation in preclinical human GBM models. Because the free drug cannot pass the blood–brain barrier (BBB), we investigated the use of nanocarriers for transport of the drug through the BBB and its efficacy when combined with radiotherapy and temozolomide (TMZ) in glioma spheroids.

**Methods:**

In vitro studies were performed using multicellular U87 human GBM spheroids. Polymeric nanocarriers (polymersomes) were loaded with MEK162. The interaction between nanocarrier delivered MEK162, irradiation and TMZ was studied on the kinetics of spheroid growth and on protein expression in the MAPK/ERK pathway. BBB passaging was evaluated in a transwell system with human cerebral microvascular endothelial (hCMEC/D3) cells.

**Results:**

MEK162 loaded polymersomes inhibited spheroid growth. A synergistic effect was found in combination with fractionated irradiation and an additive effect with TMZ on spheroid volume reduction. Fluorescent labeled polymersomes were taken up by human cerebral microvascular endothelial cells and passed the BBB in vitro.

**Conclusion:**

MEK162 loaded polymersomes are taken up by multicellular spheroids. The nanocarrier delivered drug reduced spheroid growth and inhibited its molecular target. MEK162 delivered via polymersomes showed interaction with irradiation and TMZ. The polymersomes crossed the in vitro BBB model and therewith offer exciting challenges ahead for delivery of therapeutics agents to brain tumours.

## Introduction

Glioblastoma Multiforme (GBM) is the most prevalent and deadly type of primary malignant brain tumours. Due to its cellular heterogeneity, GBM is extremely invasive and holds a fast and aggressive regression potential [1, 2]. Current treatment consists of surgery followed by radiotherapy (RT) and concomitant and adjuvant temozolomide (TMZ), with a median survival rate of 15 months [3]. Treatment optimisation for the group of GBM patients is therefore warranted. Gene expression profiling showed that more than 90% of all glioblastomas have activating mutations in the mitogen-activated protein kinase/extracellular signal-regulated kinase (MAPK/ERK) and phosphoinositide 3-kinase/Akt (PI3K/Akt) cell signaling pathways [4]. Mutations within these two pathways mostly lead to tumour formation and the most frequently dysregulated signaling cascades in human cancer are in those cell signaling pathways [5].

Binimetinib, also known as MEK162, is a potent, allosteric inhibitor of the human MEK1 and MEK2 proteins. MEK1/2 inhibition prevents activation of proteins and transcription factors that are dependent on MEK1/2 activation, which can lead to the inhibition of growth factor-mediated cell signaling resulting in inhibition of tumour cell proliferation. Previous data from our laboratory showed the MAPK inhibitor MEK162 (binimetinib) to act as radiosensitizer. The drug, when combined with fractionated irradiation, enhanced the radiation induced growth inhibition of multicellular GBM spheroids [6]. In vivo experiments, using a murine orthotopic GBM8 model, demonstrated that MEK162 additional to radiation delayed tumour growth and prolonged the survival time of the animals [6].

**Fig. 1:**
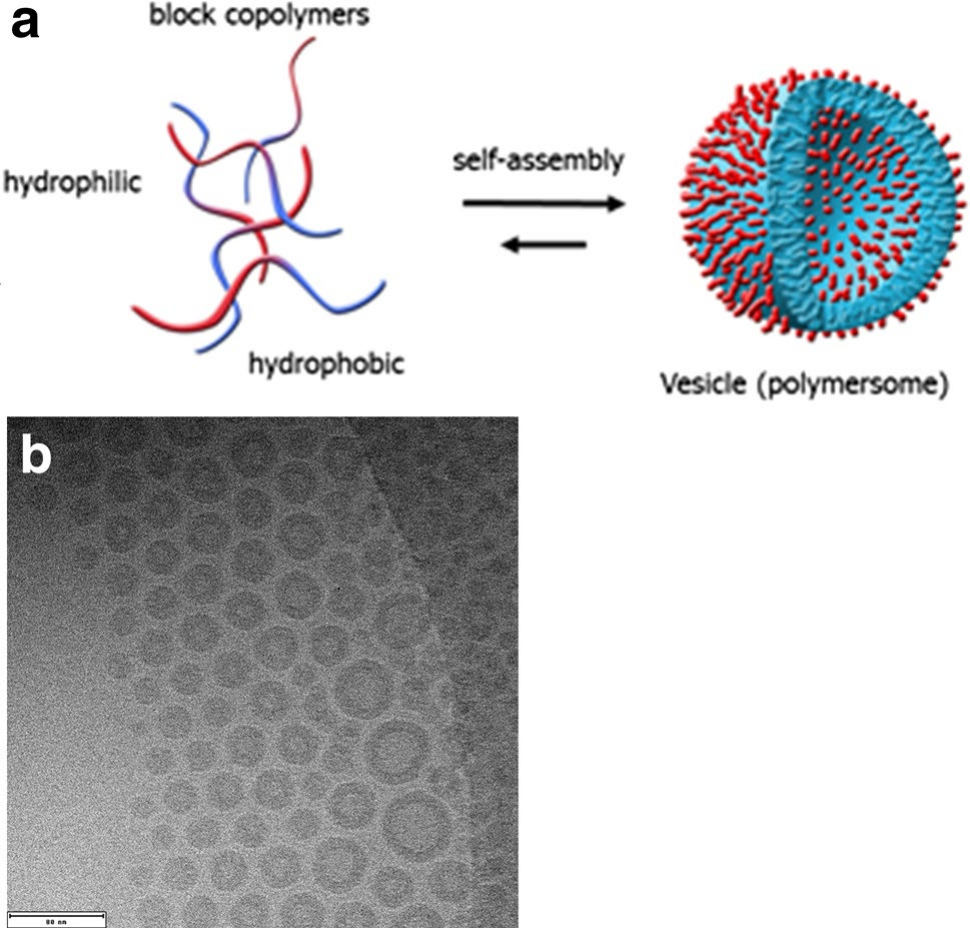
Preparation of the polymersomes. **a** Self-assembly of amphiphilic block copolymers. **b** Cryogenic transmission electron microscopic images of polymersomes

Phase 1/2 clinical trials using these targeted agents as monotherapy are ongoing, but unfortunately no breakthrough has been reported yet. A major problem is that the delivery of most chemotherapeutic and molecular targeted agents, including MAPK inhibitors, is abrogated by the presence of the blood–brain barrier (BBB) [7, 8]. For many years, different strategies have been investigated to facilitate BBB crossing of therapeutic agents [9]. A promising novel avenue is the use of nanotechnology, i.e. the use of molecular devices (nanocarriers) with a diameter ranging from 5 to 500 nm for drug delivery to the brain [10, 11]. Various nanotechnology-based approaches like micelles, liposomes, polymersomes, dendrimers and solid lipid nanoparticles have been studied and tested for glioma treatment [9, Becerril-Aragon et al., unpublished]. Liposomes have extensively been tested for drug delivery and various formulations are tested in clinical trials [12]. Polymeric carriers (polymersomes) are superior over liposomes in terms of a better loading capability, longer blood circulation time, less drug leakage and larger storage capacity [9]. Further advantages include the lower drug toxicity by controlled drug release and improved drug pharmacokinetics via increased drug stability and solubility [13]. Polymeric vesicles are characterized with an aqueous core surrounded by a hydrophobic bi-layer membrane composed of amphiphilic block copolymers (Fig. 1, [14]), allowing loading of both hydrophilic and hydrophobic agents. Polymersomes are highly versatile and biologically stable, and drug encapsulation and release capabilities can be modulated [15].

In the present study, we investigated MEK162 loaded polymeric nanocarriers in combination with irradiation and TMZ on human brain U87MG tumour cells growing as 3D spheroid. Blood–brain barrier passaging was studied in an in vitro transwell BBB model with human vascular endothelial cells.

## Materials and methods

### Cell culturing, spheroid formation and growth analysis

U87MG (Uppsala 87 Malignant Glioma) human glioma cells used in the experiments were acquired from the American Type Culture Collection (ATCC). Cells were certified mycoplasma free by regular testing (microbiome.nl). Cells were cultured at 37 °C and 5% CO_2_ and maintained as monolayer in Dulbecco’s Modified Eagle’s Medium (DMEM) supplemented with 10% Fetal Bovine Serum, 1% Penicillin/streptomycin and 2% hepes serum. Sterile phosphate buffered saline (PBS) and 0.25% Trypsin-Ethylenediaminetetraacetic acid (Trypsin–EDTA) were obtained from Gibco (Paisly, UK) dilutions. U87 spheroids were formed by plating 3000 cells/well in a transparent U-bottom 96 well cell-repellent surface plate (Cellstar®). Four days were allowed for spheroid formation before starting the treatment. Images were made with a microscope (Leica DMI3000) using the Universal Grab 6.3 software (DCILabs). The size of the spheroids was determined using the Scratch assay 6.2 software (DCILabs) and analyzed with GraphPad.

### Polymersome formation and drug loading

A drug stock solution was prepared by dissolving MEK162 in DMSO to a final a concentration of 50 µM. A polymer solution was made by dissolving 64 mg poly(butadiene-b-ethylene oxide) block copolymers (Polymer Source, Quebec, Canada) with ratio of the weight average molecular weight to the number average molecular weight (Mw/Mn) of 1.05 in 3.2 mL acetone (Fig. 1a). The final concentration was 20 mg/mL. The drug solution was added to the polymer solution and sterile PBS was added with a speed of 0.1 mL/min under 300 RPM stirring. The acetone solvent of the polymer was evaporated under nitrogen. Before experimental use, the 20 mg/mL polymersome solution was diluted 10 times, and subsequently filtered on a PD10 column (GE Healthcare) filled with Sephadex G-25. The size of the polymersomes was on average 80 nm, as confirmed by cryogenic transmission electron microscope imaging (Fig. 1b). The filtered samples were diluted 1:5 with cell culture medium for the experiments. Polymersomes were labelled with FITC ‘Isomer 1’ fluorescent dye by adding 2 µl FITC (10 mg/mL FITC in ethanol) to 1.0 mL polymersome solution. After 1 h incubation, the free FITC was removed by passing the solution through a PD10 column. For details regarding the polymersomes, see De Kruijff et al. [16].

### Cryo-TEM measurements

To determine polymersome size and morphology, they were imaged in a Jeol JEM 1400 TEM under an acceleration voltage of 120 keV. The samples were prepared by depositing 3 µL polymersome solution on a holey carbon grid (Quantifoil 2/2), which was subsequently blotted and rapidly inserted into liquid ethane. The sample was immersed in liquid nitrogen and transferred using a sample holder (Gatan model 626) to the TEM.

### Drugs and radiation

Spheroids were irradiated (5 daily fractions of 2 Gy) at room temperature using a Cobalt-60 source at a dose rate of approximately 180 Gy/h (Gammacell 220®; Atomic Energy of Canada, Mississauga, Ontario, Canada). MEK162 (Selleckchem.com) and TMZ (Sigma-Aldrich) were dissolved in DMSO with a final concentration of 1 µM and 64 µM respectively. For the combination experiments, spheroids were treated with the drugs at 1 h prior to irradiation.

### Western blot analysis

All proteins were extracted from the cells using RIPA buffer (RIPA, DNAse (1:1000), protease inhibitor cocktail (1:100), Na-orthovanadate (1:100) and PMSF (1:100)). Proteins were loaded onto 10% SDS- polyacrylamide gel electrophoresis (SDS-PAGE) for electrophoresis and then transferred to the membrane. After transfer of the proteins from the gel to the membrane, proteins were blocked with 5% BSA-PBS-Tween (0.1% Tween (Sigma-Aldrich) in PBS) primary antibody of interest was diluted in 5% BSA-PBS-Tween and added to the membrane at 4 °C overnight on a roller bench. The primary antibody was removed and the appropriated HRP conjugated secondary antibody (diluted in 5% BSA-PBS-Tween) was added to the membrane and incubated for 1 h at room temperature. The luminescence substrate was made by mixing the two components (1:1) of the Pierce ECL Plus Western Blotting Substrate kit (Product number 32132, Thermo Scientific, Rockford, USA). For ECL detection the Uvitec Cambridge Alliance 4.7 scanner was used. Proteins were stained using the Cell Signaling Technology (CST) antibodies, phospho-p44/42-ERK (#4370) (Cell Signaling #9272), phospho-H2AX (#9718) with loading control β-Actin (#3700).

### Transwell system

**Fig. 2:**
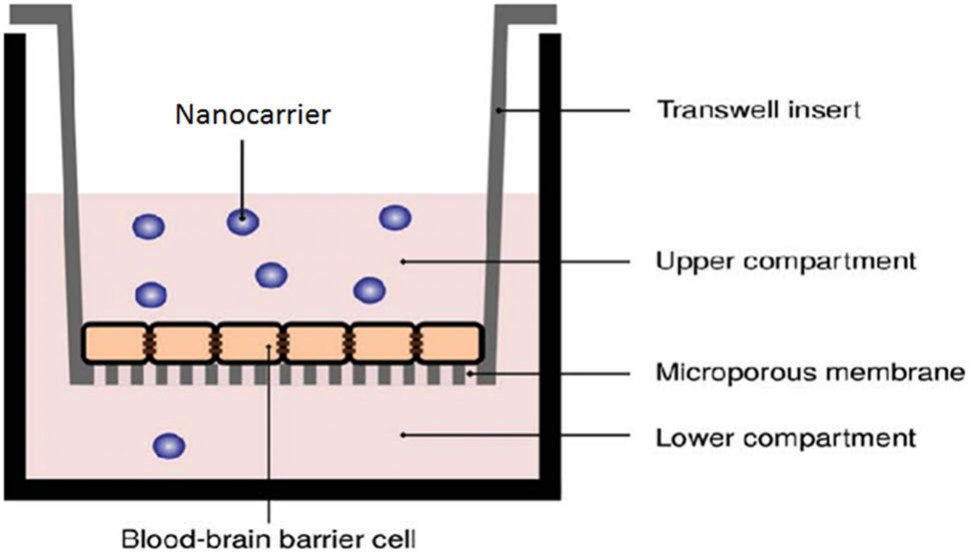
BBB transwell in vitro system. The upper and lower compartment are separated by a microporous membrane covered with a confluent layer of human cerebral microvascular endothelial (hCMEC/D3) cells

Ibidi chamber plates (Corning Transwell) with filter inserts (0.4 µm pore polycarbonate membrane) were used in the experiments. Human cerebral microvascular endothelial (hCMEC/D3) cells were grown on the microporous membrane and separated the upper and lower compartments of the transwell system (Fig. 2). Fluorescent labeled polymersomes were placed in the upper compartment, and their transport across the BBB was evaluated.

## Results

### MEK162 loaded polymersomes inhibit spheroid growth

**Fig. 3:**
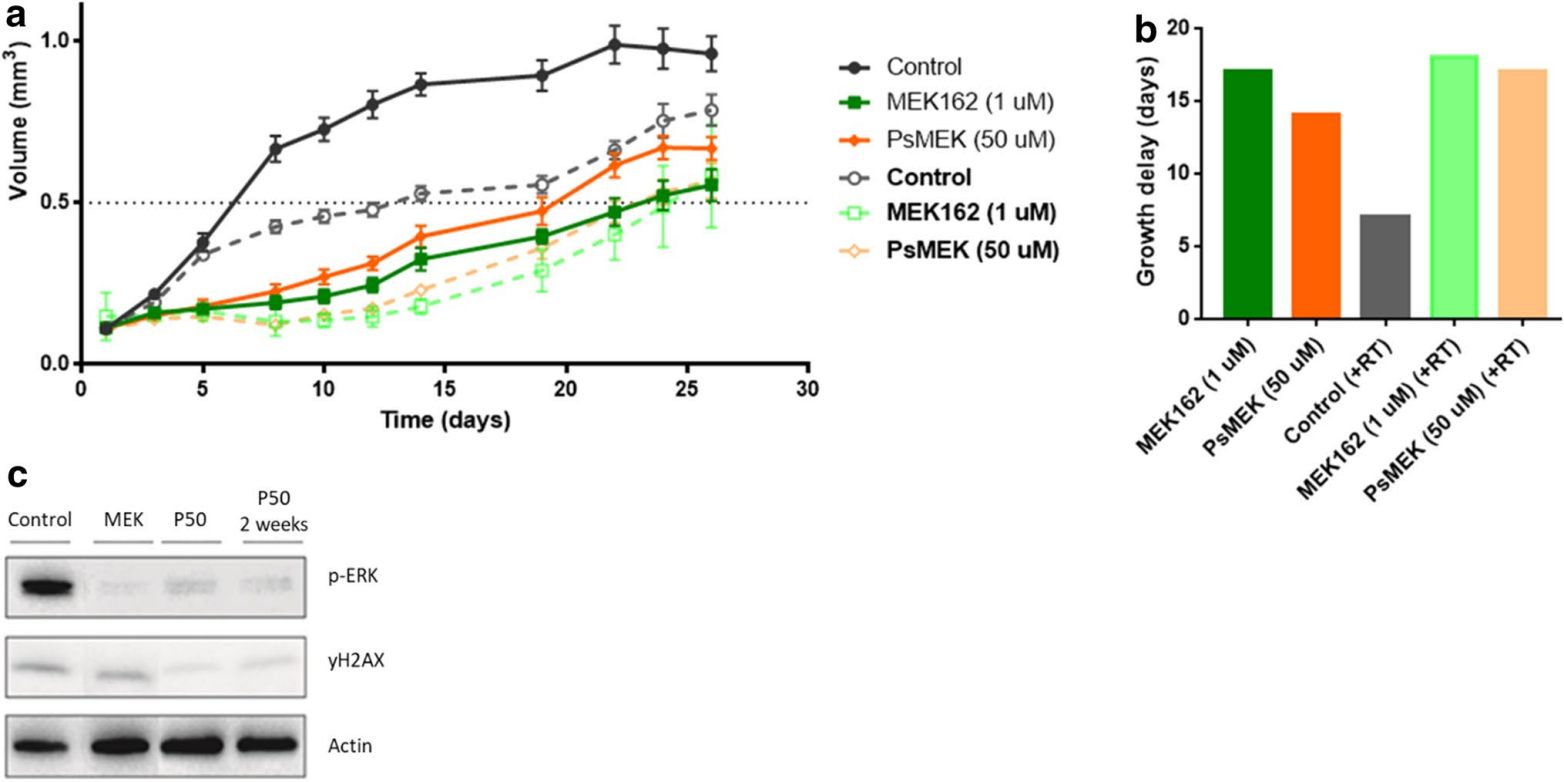
MEK162 and irradiation treatment of U87 spheroids. **a** U87 spheroid growth with 50 µM MEK162 loaded polymersomes (PsMEK) or 1 µM free MEK162 without (straight line) and with (broken line) irradiation (5 daily fractions of 2 Gy). Error bars represent mean ± SD (n = 6). **b** Growth delay, i.e. time (days) to reach V_5_ (= 5 times the start volume V_0_) between the control group and treated spheroids. **c** Western blots of U87 spheroids at day 28. P-ERK, the substrate of MEK, is reduced after exposure to either free MEK162 or MEK162 loaded polymersomes. yH2AX is a DNA double strand break marker control and actin the loading control

U87 spheroids were treated with free MEK162 (1 µM) or with MEK162 loaded polymersomes (50 µM loading concentration). Figure 3a demonstrates the volume of the spheroids over time. Both the addition of MEK162 loaded polymersomes as well as free MEK162 delayed the spheroid growth relative to controls. Further delay in spheroid growth was observed in combination with fractionated irradiation (5 daily fractions of 2 Gy). Growth delay times are displayed in Fig. 3b, and show similarity between MEK162 applied as free drug or encapsulated in polymersomes, with and without irradiation. Also, p-ERK protein expression was reduced both in spheroids treated with free MEK162 and with MEK162 loaded polymersomes (Fig. 3c).

### Combination therapy of MEK162 loaded polymersomes with TMZ and irradiation

**Fig. 4:**
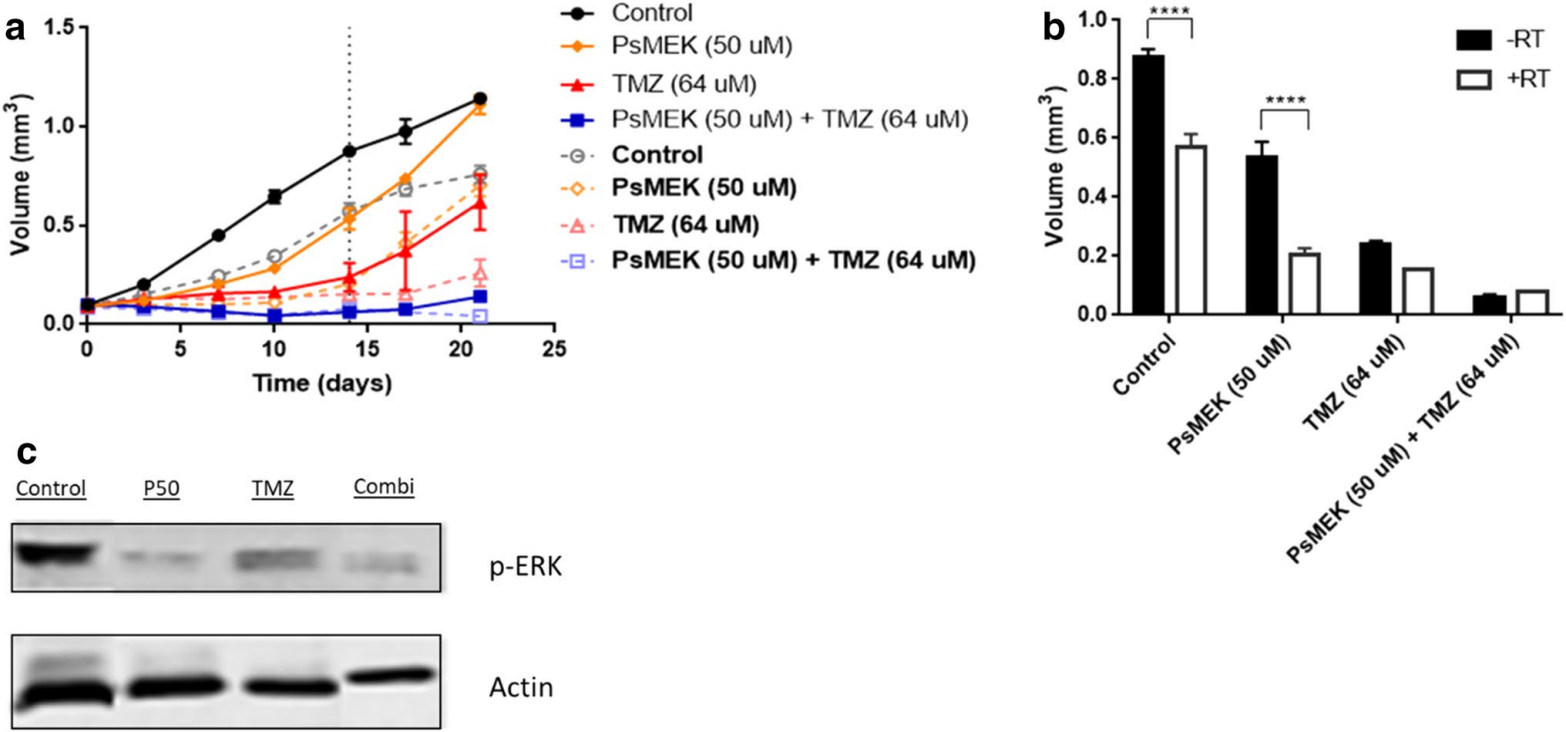
Synergistic effect when combining MEK162 loaded polymersomes with TMZ. **a** Volume growth of the U87 spheroids treated with different conditions (insert) without (straight line) or with (broken line) irradiation (5 daily fractions of 2 Gy). Spheroids were treated with 50 µM MEK162 loaded polymersomes (PsMEK), 64 µM TMZ and their combination. **b** Volume of spheroids at day 14 (vertical black broken line in (**a**) after treatment with and without fractionated irradiation. Two-way ANOVA was used to see if there is a significant difference in one group when using fractionated radiation or not (****p < 0,0001). **c** Western blots of U87 spheroids at day 21 that were exposed to 50 µM MEK162 loaded polymersomes or TMZ or their combination. The expression of p-ERK is reduced after exposure to MEK162 loaded polymersomes alone and when combined with TMZ. Actin is loading control

Figure 4a shows spheroid growth after treatment with MEK162 loaded polymersomes, TMZ, irradiation and its combinations. Spheroid volumes at day 15 after the start of treatment, with and without irradiation are displayed in Fig. 4b. The data shows that radiation alone (5 daily fractions of 2 Gy) reduced the spheroid volume. The combination with MEK162 loaded polymersomes significantly enhanced the volume reduction. Protein expression data revealed a downregulation of p-ERK following exposure to MEK162 polymersomes alone and when combined with TMZ (Fig. 4c).

### Polymersomes are able to pass the in vitro BBB

**Fig. 5:**
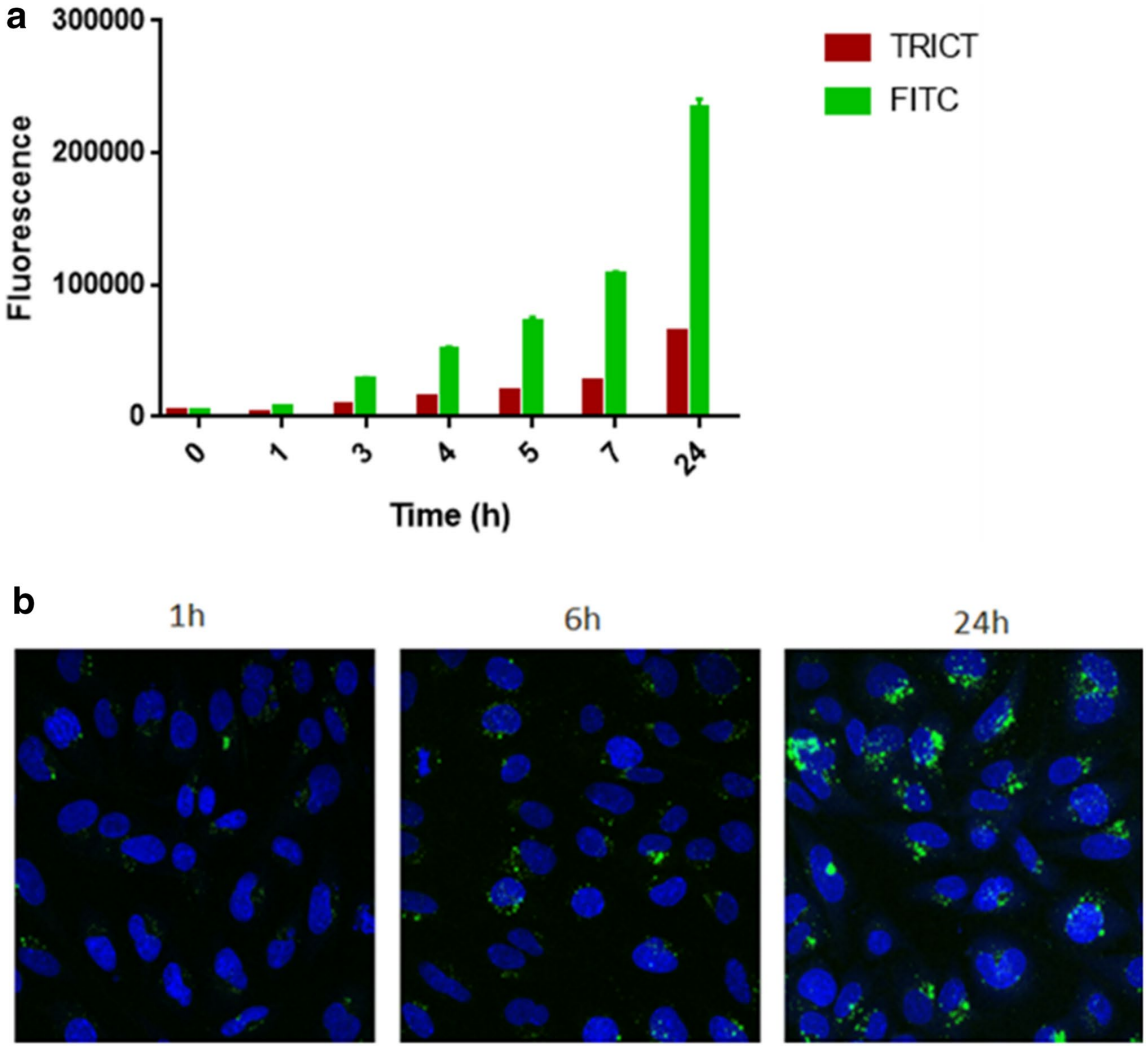
Fluorescent labelled polymersomes were added to the upper compartment and passaging was measured via visualisation in the lower compartment. **a** Kinetics of transport of FITC fluorescent polymersomes (green) and control fluorescent compound TRITC (red) towards the lower compartment. X-axis: time (hours); Y-axis fluorescence. Error bars represent mean ± SD (n = 4). **b** uptake of polymersomes in the hCMEC/D3 cells at 1 h, 6 h, and 24 h.; Nuclei (DAPI; blue staining); nanocarriers (FITC, green) (× 200)

Polymersomes were labelled with the fluorescent dye FITC. TRITC, another fluorescent compound, was used as a control. Results show that the fluorescence signal in the lower compartment increased over time (Fig. 5a), reaching a plateau at 24 h. Figure 5b illustrates that the uptake of the fluorescent labeled polymersomes by the human cerebral microvascular endothelial cells increases in time from 1, 6 and 24 h after loading of the upper compartment, consistent with the increased transport towards the lower compartment of the BBB model.

## Discussion

The present data show that the allosteric MAPK inhibitor MEK162, either applied as free drug or encapsulated in polymeric nanocarriers inhibited the growth of U87 multicellular glioma spheroids and reduced the expression of downstream p-ERK protein. Synergy was found between MEK162 loaded nanocarriers and irradiation on the endpoint spheroid volume, while TMZ had a further additive effect. No antagonism between the three treatment modalities was found. Transwell system experiments (cf. Figs, 2, 4) showed passaging of the nanocarriers over the BBB.

Despite high drug loading efficacy of the nanocarriers (~ 40% at 50 µM loading concentration), the biological efficacy of encapsulated MEK162 in polymersomes was low compared with the free drug: 1 µM free MEK162 caused a large inhibition of spheroid growth, while MEK162 delivered via the nanocarriers was less effective. Previously, we showed that free MEK162 was synergistic to radiation [6], and this was not confirmed in the present study on the endpoint spheroid growth delay, with MEK162 delivered via polymersomes (cf. Fig. 3), indicating that a cellular drug concentration for a radiosensitizing effect was not reached. This might either be due to a low intracellular release of the drug from the nanocarriers or a dilution effect in the cytoplasm, or a combination of both. Detailed information about the drug binding capacity as well as the efficacy and kinetics of drug release of the polymersomes and of other types of nanocarriers, is warranted. A thorough understanding of the cellular distribution of the nanocarriers and interactions with cellular structures is a key issue in drug carrier systems due to the direct correlation that exists between cellular uptake, intracellular trafficking mechanism, and drug bioavailability, clinical efficacy, and therapeutic outcome of the entrapped therapeutic compound [17]. In a previous study, we reported on the kinetics of the uptake of fluorescently labeled polymersomes in U87 spheroids [16]. In about 4 days, the polymersomes have diffused nearly throughout the spheroids. After 1 week, the distribution was found to be completely homogeneous. Hence, for the present long term experiments, nanocarriers will have been uniformly distributed over the spheroid volume. In vivo studies on nanocarrier distribution in healthy tissues and tumours are emerging [e.g. 18].

Regarding the combination treatment of MEK162 and TMZ, our data indicate an additive effect of both compounds. The TMZ dose we used in our study was relatively low, consistent with the negative MGMT promotor methylation status and therewith related sensitivity to TMZ of U87 cells [19]. The additive effect rather than either an antagonistic or synergistic effect most likely can be found in the different mechanism of action, i.e. direct DNA damage induction by the methylating drug TMZ and inhibition of the MAPK signaling pathway by MEK162. Mechanistically, MAPK inhibition leads to down-regulation and dephosphorylation of the cell cycle checkpoint proteins CDK1/CDK2/WEE1 and of the DNA damage response proteins p-ATM/p-CHK2, finally inhibiting cell proliferation, which was demonstrated in a previous report from our laboratory [6].

The efficacy of inhibition of MEK1/2 by MEK162 was verified via western blot analysis of the phosphorylation of the downstream in the pathway Erk1/2 protein. The analysis revealed a larger inhibition of p-ERK in samples taken from U87 spheroids than from U87 cells growing in monolayer (data not shown), confirming the superiority of using 3D model systems in preclinical studies [20]. Currently, MEK inhibitors are in clinical trials, in particular for RAS or BRAF mutated tumours. Binimetinib has gained global approval in the USA, but only for patients with a BRAF mutation and when combining it with the BRAF inhibitor encorafenib [21]. There is cross talk between the MAPK and PI3K pathways, because both pathways start with activation of the tyrosine kinase receptor [22]. In this respect, the polymeric nanocarriers are an interesting option, because they can be loaded with dual or even multiple drug combinations, e.g. inhibitors of both the MAPK and the PI3K pathways.

The 80 nm diameter sized fluorescent labeled polymeric nanocarriers were found to cross the layer of human cerebral microvascular endothelial cells, as a surrogate BBB, towards the lower compartment of the transwell system (cf. Figure 5). To further increase the polymersome delivery to the brain tumour location, conjugation of the nanocarriers with proteins that are specific or over-expressed in the BBB and not in other body tissues—in order to increase local delivery—and/or specific for the brain tumour target, is a most promising option. Several candidates have been proposed, including glutathione and transferrin. Glutathione interacts with proteins located in the brain that are involved in the transport of molecules across the BBB [11]. Glutathione-coated docetaxel-loaded PEG-PLGA nanoparticles were studied in an in vitro BBB permeation model and on their cytotoxic efficacy in rat glioma cells by Grover et al. [23]. They showed that docetaxel loaded nanoparticles were able to pass the BBB better than the free drug solution. Also, the docetaxel delivered via glutathione conjugated nanoparticles was significantly more cytotoxic than the free drug. Mapanao et al. [24] studied the use of peptide functionalized passion fruit-like nanoarchitectures. They demonstrated in 3D pancreatic carcinoma spheroids that both the targeting via the transferrin receptor and internalization of the nanoparticles were increased.

Taken together, the polymeric nanocarriers are multifunctional and might be used in the treatment of different diseases including cancer for drug transport. Most challenging is the feasibility of conjugation of the nanocarriers with specific proteins, to facilitate their uptake in the desired site of therapeutic interaction while reducing adverse side effects to the normal tissues [25]. In particular, the combination of *fractionated* irradiation (60 Gy in 30 fractions of 2 Gy, 5 fractions per week for 6 weeks in GBM patients) with other therapeutic agents, controlled slow release of the radiosensitizing agents in order to exploit the typical features of radiation on cells and tissues, the so-called 5 ‘Rs’ or hallmarks of radiobiology, is a very promising approach [26]. The present data represents the first steps into that direction.

In conclusion, MEK162 loaded polymeric nanocarriers are taken up by multicellular spheroids, reduce their growth, inhibit the molecular target and interact with irradiation and TMZ. A large number of issues regarding nanocarrier drug loading, drug release, stability in tissue fluids and directed delivery to the target cells and tissue are currently under investigation. Nevertheless, the nanocarrier approach offers exciting challenges ahead for delivery of therapeutics agents to GBM patients.
